# Intravenous melatonin abuse leading to recurrent aortic valve endocarditis: a case report and discussion

**DOI:** 10.1186/s12888-016-0754-4

**Published:** 2016-02-24

**Authors:** Mark B. Warren, Jonathan Stabler, Melissa M. Hagman

**Affiliations:** Department of Psychiatry, University of Washington, Boise Veterans Administration Medical Center, 500 W. Fort St., Boise, ID 83702 USA; Department of Internal Medicine, University of Washington, Boise Veterans Administration Medical Center, 500 W. Fort St., Boise, ID 83702 USA

**Keywords:** Infective endocarditis, Melatonin abuse, Melatonin injection, *Granulicatella adiacens*

## Abstract

**Background:**

Reports of inappropriate medication use are widespread. There is a growing literature detailing abuse of drugs not typically thought to have high abuse liability. Melatonin is considered to be generally safe and is categorized by the Food and Drug Administration as a nutritional supplement. There are no known reports of intravenous melatonin abuse in the medical literature.

**Case presentation:**

The authors report a case of a patient injecting melatonin with euphoric and then sedative effects leading to two episodes of infective endocarditis culminating in aortic valve replacement.

**Conclusion:**

Infective endocarditis continues to be a major potential complication of intravenous drug abuse. The proliferation of novel street drugs, resurgence in the use of older drugs and ongoing abuse of medications warrant continued research and vigilance in treating substance use disorders and attendant medical complications.

## Background

The changing landscape of drug use, including continued innovation of street drugs [1], intravenous injection of tablets meant for oral ingestion [2] and a changing demographic and resurgence in abuse of older drugs such as heroin [3], present a continuing challenge to healthcare teams. Prescription opioid and benzodiazepine abuse is a widely known problem but there is a growing literature describing abuse of medications that are not conventionally considered to have high abuse liability including several types of antidepressants [4]. Intravenous injection of tablets can lead to complications such as infective endocarditis (IE), stroke and a variety of pulmonary pathology [5].

We report a case of a patient injecting melatonin tablets leading to IE initially with *Granulicatella adiacens* and subsequently with *Streptococcus salivarius* (*viridans*) necessitating aortic valve replacement. Melatonin is thought to be generally safe and is categorized by the FDA as a dietary supplement. It is a neurohormone produced by the pineal gland and thought to be important in the maintenance of the sleep-wake cycle. It is most commonly used for general sleep disorders, jet lag and other circadian rhythm disorders and has also been reported to have cytostatic action on some human cancers at doses typically between 1.5 mg and 10 mg at night with variable efficacy. Common side effects include fatigue, somnolence and hypothermia. There are no known absolute contraindications to its use but caution is advised with hepatic or renal impairment or immune disorders [6]. It has not been reported to have high abuse liablity, nor reported to cause euphoria. To our knowledge this is the first report of its kind in the literature.

## Case presentation

Mr. S is a 58-year-old gentleman with a complicated medical history including recurrent pulmonary embolism related to antiphospholipid syndrome (APS), pulmonary arteriovenous malformation, major depression with two prior deliberate overdose attempts and remote intravenous drug abuse (IVDA). He initially presented to a community hospital following a motor vehicle accident after new onset seizures and was found to have a small intraparenchymal brain hemorrhage. Anticoagulation was held and he recovered with no long-term neurologic sequelae.

One month later he was admitted to inpatient medicine with suicidal ideation and hyponatremia, stabilized and transferred to psychiatry. Later exam revealed a weight increase of 2.3 kg over a 48 h period along with low grade fever, new systolic and diastolic murmurs, significant bilateral lower extremity edema and recurrence of hyponatremia. Blood cultures grew *Granulicatella adiacens* and trans-esophageal echocardiogram showed aortic valve vegetations and moderate to severe regurgitation. He was treated for endocarditis with penicillin and gentamicin. The infection was attributed to poor dentition (Mr. S adamantly denied any IVDA) and three teeth were extracted. He was referred for valve replacement but this was postponed due to concern over bleeding risk.

Over the next several months, Mr. S. had multiple admissions to inpatient psychiatry for suicidality and a medical admission for pulmonary embolism and worsening biventricular heart failure. He was referred again for valve replacement.

**Fig. 1:**
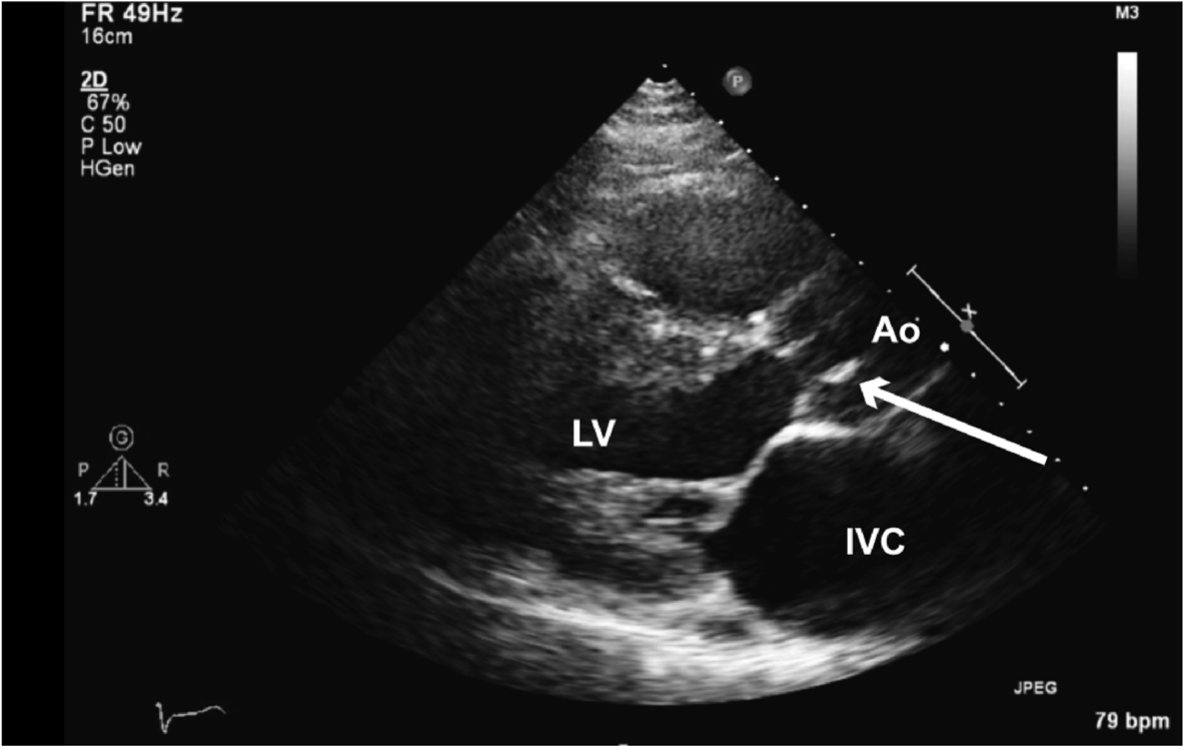
Echocardiogram showing mobile aortic valve vegetation

Prior to surgery he presented with increasing malaise, fevers and chills. Blood cultures grew *Streptococcus salivarius* (*viridans*) and examination showed worsening heart failure, mitral and aortic insufficiency murmurs, splinter hemorrhages on two fingers and a Janeway lesion on the left distal forearm. Additionally, he had a palpable venous cord with overlying injection marks on the right lateral wrist. Echocardiogram showed aortic valve endocarditis with mobile vegetation (Fig. 1) and severe regurgitation. He was treated with targeted intravenous antibiotics.

During this admission Mr. S reported he had been crushing single 5 mg tablets of melatonin, mixing with ice water and injecting intravenously several times per week, up to two times per day for the past two years. He confirmed this history on psychiatric consultation and added that he now had a sense of relief about having “a clean slate, no more secrets.” Mr. S. described an initial euphoric effect with melatonin injection. This diminished but he continued injecting to help initiate sleep.

Mr. S underwent aortic valve replacement a week after he disclosed to staff his abuse of melatonin. Of note, he had only one psychiatric hospitalization in the nine months subsequent to his valve replacement compared with six inpatient psychiatric admissions in the year prior to his surgery.

## Discussion

Melatonin is thought to be generally safe with no reported abuse liability and its prescription drug counterpart, ramelteon, a melatonin receptor agonist, is also thought to have low to no abuse potential [7]. Intravenous injection of any substance, however, places individuals at risk for infectious and embolic complications and continues to be a source of worldwide medical and public health attention [8]. Mr. S’s complications from melatonin injection included recurrent aortic valve endocarditis and we hypothesize that his initial stroke may have been due to tablet material traveling through his known pulmonary arteriovenous malformation. These types of embolic phenomena have been reported with injection of other types of tablets [9]. It must be noted that his recurrent endocarditis was most likely related to injection habits and nonsterile technique as opposed to any melatonin-specific characteristics.

IE remains a serious illness with high morbidity and mortality [10]. Its approximate incidence is 3 to 9 cases per 100,000 in industrialized nations. The precise incidence of IE in IVDA is not known but remains problematic [8]. It is estimated that up to 41 % of IVDA patients with bacteremia will develop IE [11].

Offending organisms in IE are most often streptococci and staphylococci [12]. IE caused by *Granulicatella adiacens* is thought to be a rare but increasingly frequent cause and can be associated with severe cases of bacteremia regardless of prior immune state. A case series and literature review from early 2015 identified only 29 published cases of IE due to *G. adiacens* and infection carried a 17% mortality rate. Forty-four percent of cases involved the aortic valve [13].

## Conclusions

We report the first known case of recreational melatonin injection leading to IE with *Granulicatella adiacens* and subsequently with *Streptococcus salivarius* (*viridans*) necessitating aortic valve replacement. IE continues as a major potential complication of IVDA. The proliferation of novel street drugs, resurgence in the use of older street drugs and ongoing abuse of prescribed medications and dietary supplements warrant continual research and vigilance in treating substance use disorders and attendant medical complications.

### Consent

Written consent for publication in the medical literature to include pertinent imaging was obtained from the patient.

## Abbreviations

IE: Infective endocarditis
IVDA: Intravenous drug abuse
APS: Antiphospholipid syndrome
FDA: Food and Drug Administration
